# Genomic Organization of Repetitive DNA Elements and Extensive Karyotype Diversity of Silurid Catfishes (Teleostei: Siluriformes): A Comparative Cytogenetic Approach

**DOI:** 10.3390/ijms20143545

**Published:** 2019-07-19

**Authors:** Sukhonthip Ditcharoen, Luiz Antonio Carlos Bertollo, Petr Ráb, Eva Hnátková, Wagner Franco Molina, Thomas Liehr, Alongklod Tanomtong, Costas Triantaphyllidis, Catherine Ozouf-Costaz, Sampan Tongnunui, Puan Pengseng, Weerayuth Supiwong, Rouben Aroutiounian, Marcelo de Bello Cioffi

**Affiliations:** 1 Toxic Substances in Livestock and Aquatic Animals Research Group, Department of Biology, Faculty of Science, Khon Kaen University, Muang, Khon Kaen 40002, Thailand; 2 Departamento de Genética e Evolução, Universidade Federal de São Carlos (UFSCar), Rodovia Washington Luiz Km. 235, C.P. 676, São Carlos, SP 13565-905, Brazil; 3 Laboratory of Fish Genetics, Institute of Animal Physiology and Genetics, Czech Academy of Sciences, Rumburská 89, Liběchov 277 21, Czech Republic; 4 Department of Zoology and Fisheries, Faculty of Agrobiology, Food and Natural Resources, Czech University of Life Sciences, Kamýcká 129, Prague 165 00, Czech Republic; 5 Departamento de Biologia Celular e Genética, Centro de Biociências, Universidade Federal do Rio Grande do Norte (UFRN), Natal, RN 59078970, Brazil; 6 Institute of Human Genetics, University Hospital Jena, Jena 07747, Germany; 7 Department of Genetics, Development and Molecular Biology, Faculty of Sciences, School of Biology, Aristotle University of Thessaloniki, University Campus, Thessaloniki 54124, Greece; 8 Laboratorie Evolution Paris Seine, Institut de Biologie Paris Seine (IBPS), Sorbonne Universités, Case 5, 7 Quai St Bernard, Paris, 75952 Paris CEDEX 05, France; 9 Department of Conservation Biology, Mahidol University, Kanchanaburi Campus, Sai Yok, Kanchanaburi Province 71150, Thailand; 10 School of Agricultural of Technology, Walailak University, Thasala, Nakhon Si Thammarat 80160, Thailand; 11 Faculty of Applied Science and Engineering, Khon Kaen University, Nong Khai Campus, Muang, Nong Khai 43000, Thailand; 12 Department of Genetics and Cytology, Yerevan State University, Yerevan 0025, Armenia

**Keywords:** fish cytotaxonomy, chromosome banding, FISH, rDNA classes, CGH

## Abstract

The catfish family Siluridae contains 107 described species distributed in Asia, but with some distributed in Europe. In this study, karyotypes and other chromosomal characteristics of 15 species from eight genera were examined using conventional and molecular cytogenetic protocols. Our results showed the diploid number (2*n*) to be highly divergent among species, ranging from 2*n* = 40 to 92, with the modal frequency comprising 56 to 64 chromosomes. Accordingly, the ratio of uni- and bi-armed chromosomes is also highly variable, thus suggesting extensive chromosomal rearrangements. Only one chromosome pair bearing major rDNA sites occurs in most species, except for *Wallago micropogon*, *Ompok siluroides*, and *Kryptoterus giminus* with two; and *Silurichthys phaiosoma* with five such pairs. In contrast, chromosomes bearing 5S rDNA sites range from one to as high as nine pairs among the species. Comparative genomic hybridization (CGH) experiments evidenced large genomic divergence, even between congeneric species. As a whole, we conclude that karyotype features and chromosomal diversity of the silurid catfishes are unusually extensive, but parallel some other catfish lineages and primary freshwater fish groups, thus making silurids an important model for investigating the evolutionary dynamics of fish chromosomes.

## 1. Introduction

The family Siluridae is a lineage of freshwater catfishes widely distributed through Eurasia, but with the highest diversity in South and Southeast Asia [1]. It comprises 107 recognized species [2], some of them also distributed in temperate regions, such as *Silurus glanis*, *S. aristotelis*, *S. soldatovi,* and *S. asotus* [3,4,5,6,7]. The phylogenetic position of this family remains not well resolved [8,9,10,11,12,13], although morphological and molecular data have confirmed its monophyletic status within Siluriformes [3,8,9].

Siluridae catfishes represent one of the most interesting fish groups from a 2*n* evolutionary point of view, owing to their wide distribution, unique ecological niche, and known evolutionary trajectory [7]. Siluridae includes one of the largest freshwater fish species—*Silurus glanis*—which commonly reaches 2 m in size and over 300 kg in weight [14], and is highly valued in the food market [15]. Several other species, such as *Micronema cheveyi*, *Phalaconotus apogon*, *P. bleekeri*, *Wallago attu*, and *W. micropogon*, also comprise important food sources [16] or are ornamental fishes, like the glass catfish *Kryptopterus bicirrhis* [17].

Cytogenetic studies have proven useful to discover and explore cryptic biodiversity in a number of fish groups [18]. Particularly, in complex and ecologically diverse fish groups, cytogenetic studies have made important contributions to elucidate the evolutionary pathways of distinct fish groups, owing to their particular chromosomal and genomic characteristics [19]. In fact, these approaches can reveal a set of characters usually not accessible by other research methods, thus refining evolutionary investigations [19,20]. Particularly, repetitive DNA sequences, which constitute the major component of the eukaryotic genome, have enormous potential for expanding the knowledge of karyotype differentiation [21]. In addition, the recent use of the comparative genomic hybridization (CGH) has allowed deeper analyses of fish genome organization at the chromosomal level by comparing closely related species [22,23,24,25].

Among the silurid catfishes, chromosomal studies are often restricted to conventional protocols to determine the diploid number (2*n*) and karyotype composition. Molecular cytogenetic approaches (e.g., chromosomal mapping of rDNA sequences) have been done in only two species of the genus *Ompok* [26]. Up to date, only 24 species from 8 of the 13 recognized genera had been cytogenetically examined (Table 1). The overall data show that the chromosome number varies from 2*n* = 28 in *Silurus microdorsalis* [27] to 2*n* = 92 in *Kryptopterus cryptopterus* [28].

Here, we add new chromosomal data for several silurid species from different genera, some of them analyzed for the first time, as well as others re-analyzed by different procedures (Table 1). We aimed to assess their karyotype structure, rDNA distribution, and interspecific genomic divergences through CGH experiments. The results added new informative characters useful in comparative genomics at the chromosomal level and highlighted extensive karyotype diversity among the analyzed species.

## 2. Results

### 2.1. Standard Karyotypes

**Figure 1:**
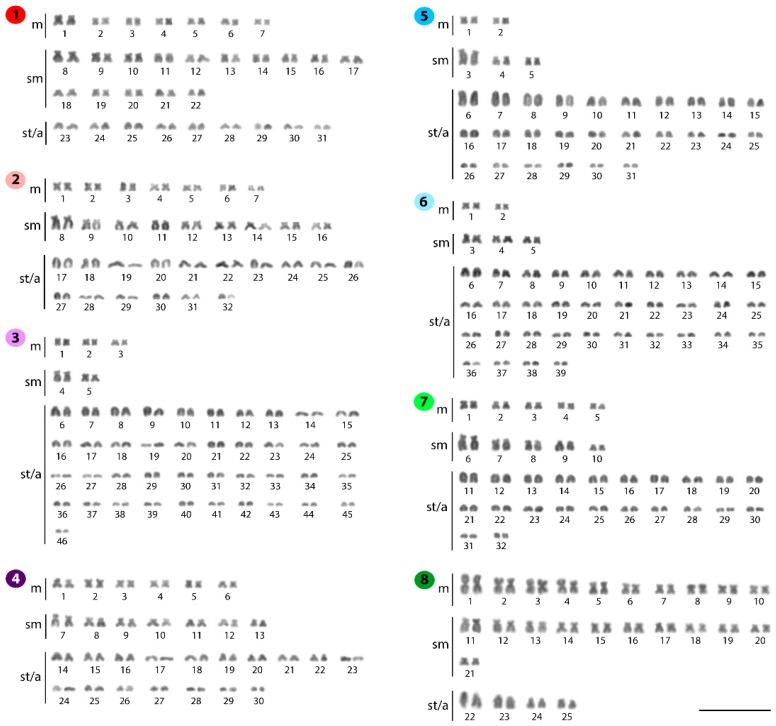
Karyotypes of *Belodontichthys truncates* (**1**); *Kryptopterus bicirrhis* (**2**); *Kryptopterus geminus* (**3**); *Kryptopterus limpok* (**4**); *Kryptopterus macrocephalus* (**5**); *Micronema cheveyi* (**6**); *Ompok fumidus* (**7**); and *Ompok siluroides* (**8**) arranged following Giemsa-staining. Bar = 5 μm.

**Figure 2:**
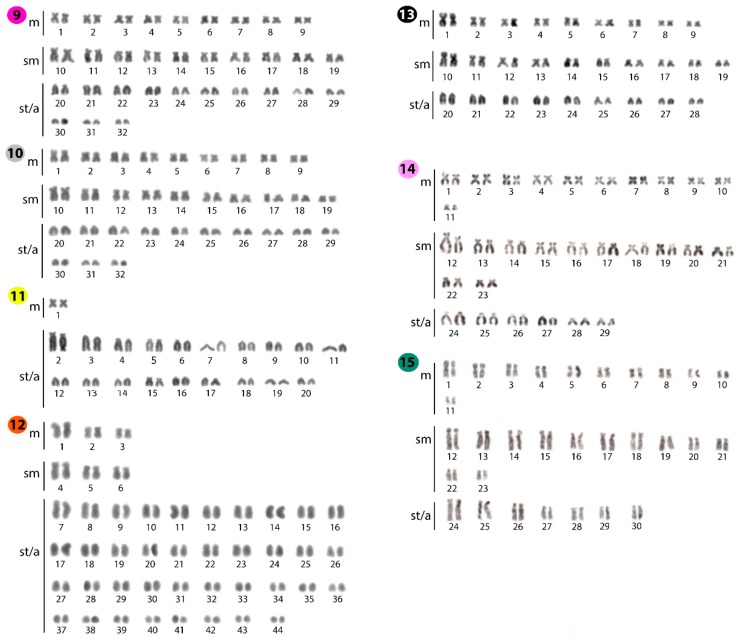
Karyotypes of *Phalacronotus apogon* (**9**); *Phalacronotus bleekeri* (**10**); *Silurichthys phaiosoma* (**11**); *Wallago attu* (**12**); *Wallago micropogon* (**13**); *Silurus aristotelis* (**14**); and *Silurus glanis* (**15**) arranged following Giemsa-staining. Bar = 5 μm.

The diploid number of the 15 species analyzed varied from 40 (*Silurichthys phaiosoma*) to 92 chromosomes (*Kryptopterus giminus*). Substantial 2*n* variation occurs even among congeneric species. The only exception was observed for both *Phalacronotus* species, *P. apogon,* and *P. bleekeri*, which shared the same 2*n* and karyotype structures (i.e., 2*n* = 64, 9m+10sm+13st/a). In all species, no numerical or structural polymorphism between the sexes was observed, thus there was no evidence of differentiated sex chromosomes (Figure 1 and Figure 2).

### 2.2. Fluorescence In Situ Hybridization (FISH)-Mapping

**Figure 3:**
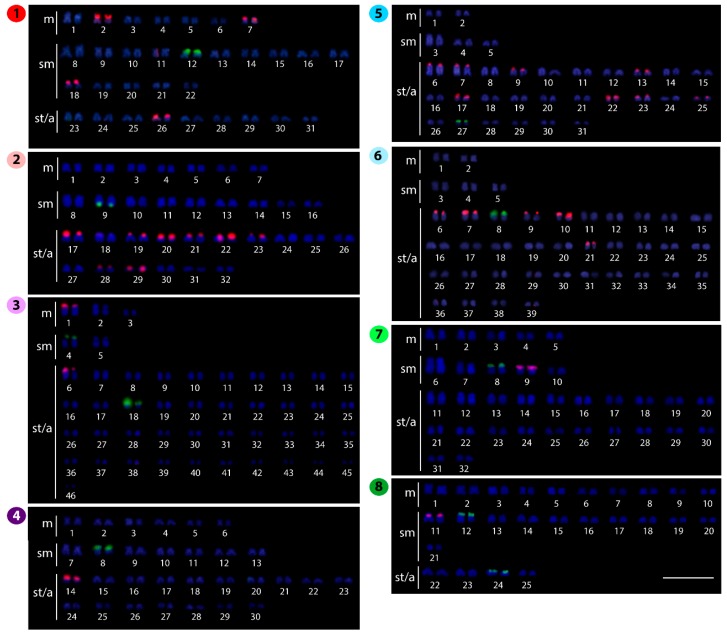
Karyotypes of *Belodontichthys truncates* (**1**); *Kryptopterus bicirrhis* (**2**); *Kryptopterus geminus* (**3**); *Kryptopterus limpok* (**4**); *Kryptopterus macrocephalus* (**5**); *Micronema cheveyi* (**6**); *Ompok fumidus* (**7**); and *Ompok siluroides* (**8**) arranged from chromosomes after double-fluorescence in situ hybridization (FISH) with 5S rDNA (red) and 18S rDNA (green) probes. Bar = 5 μm.

**Figure 4:**
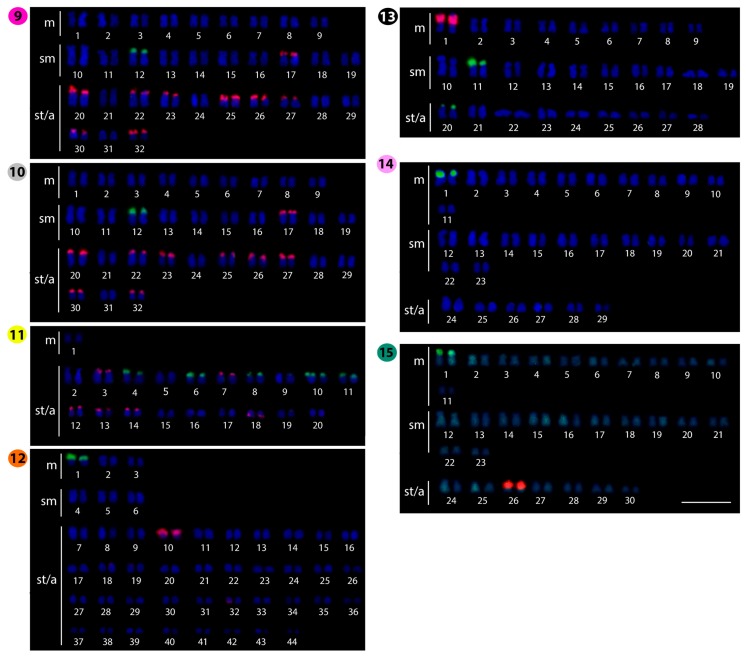
Karyotypes of *Phalacronotus apogon* (**9**); *Phalacronotus bleekeri* (**10**); *Silurichthys phaiosoma* (**11**); *Wallago attu* (**12**); *Wallago micropogon* (**13**); *Silurus aristotelis* (**14**); and *Silurus glanis* (**15**) arranged from chromosomes after double-FISH with 5S rDNA (red) and 18S rDNA (green) probes (except for *S. aristotelis*, where only 18S rDNA is indicated). Bar = 5 μm.

**Figure 5:**
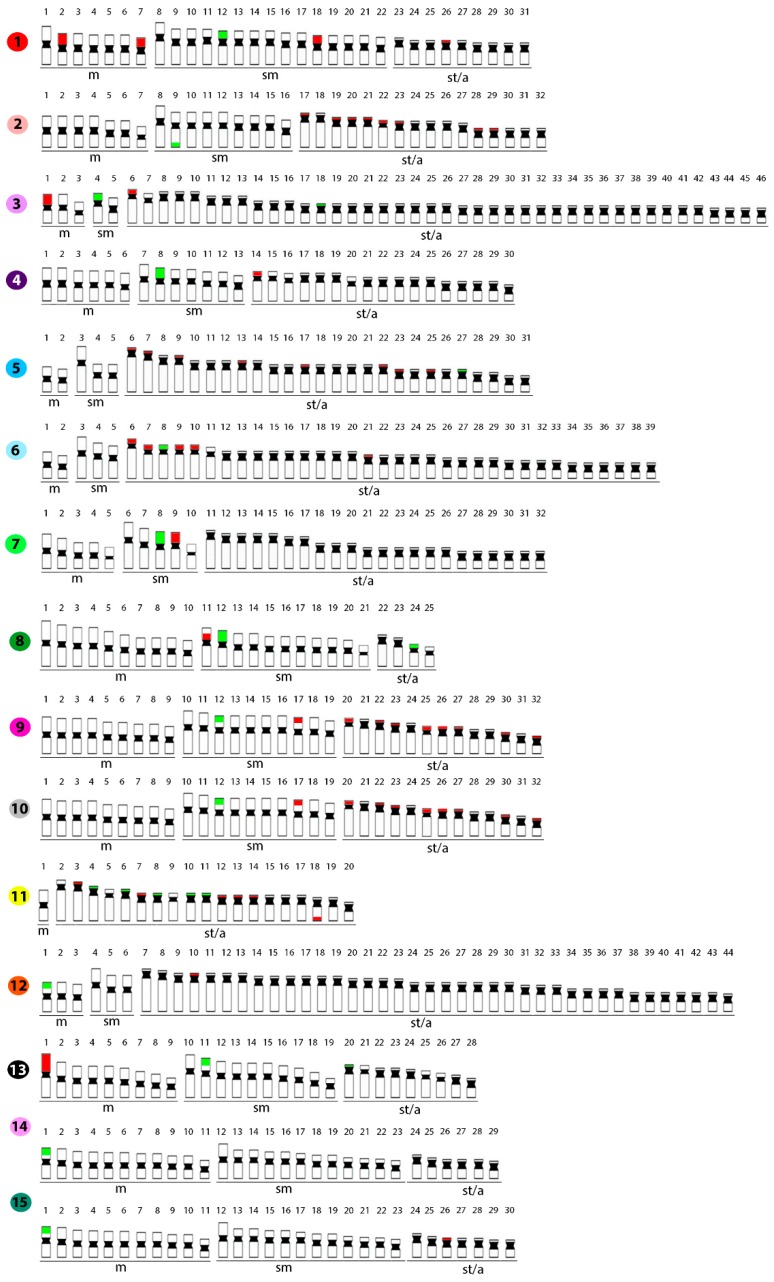
Representative idiograms of *Belodontichthys truncates* (**1**); *Kryptopterus bicirrhis* (**2**); *Kryptopterus giminus* (**3**); *Kryptopterus limpok* (**4**); *Kryptopterus microcephalus* (**5**); *Micronema cheveyi* (**6**); *Ompok fumidus* (**7**); *Ompok siluroides* (**8**); *Phalacronotus apogon* (**9**); *Phalacronotus bleekeri* (**10**); *Silurichthys phaiosoma* (**11**); *Wallago attu* (**12**); *Wallago micropogon* (**13**); *Silurus aristotelis* (**14**); and *Silurus glanis* (**15**), showing the distribution of the 5S (red) and 18S (green) rDNA sites on the respective chromosomes (except for *S. aristotelis*, where only 18S rDNA is indicated).

The 18S rDNA probe hybridized to only one chromosomal pair in most species, namely *B. truncates, K. limpok*, *K. macrocephalus*, *M. cheveyi*, *O. fumidus*, *P. apogon, P. bleekeri*, and *W. attu*. This site is located in the subtelomeric/telomeric region of the short arms of that chromosome pair in all species, except for *K. bicirrhis*, in which it is located in the telomeric region of the long arms. Exceptions for this frequent pattern are *K. geminus*, *O. siluroides,* and *W. micropogon*, in which two chromosome pairs bear 18S rDNA genes and *S. phaiosoma* with five chromosome pairs (Figure 3, Figure 4 and Figure 5).

In contrast, the 5S rDNA sites showed a large variation in distribution, ranging from one chromosome pair in *K. limpok, O. fumidus*, *O. siluroides*, *W. attu,* and *W. micropogon,* up to six pairs in *K. geminus*, *B. truncates*, *M. cheveyi*, and *S. phaiosoma*. In addition, four other species had remarkably increased numbers of chromosomes displaying such sites, namely *K. bicirrhis* and *K. macrocephalus* with eight, and *P. apogon* and *P. bleekeri* with nine chromosome pairs (Figure 3, Figure 4 and Figure 5). Fluorescence in situ hybridization (FISH) using the (TTAGGG)*_n_* telomeric probe revealed hybridization signals on telomeres of all chromosomes of *S. phaiosoma* (Figure S1).

### 2.3. Comparative Genomic Hybridization (CGH)

**Figure 6:**
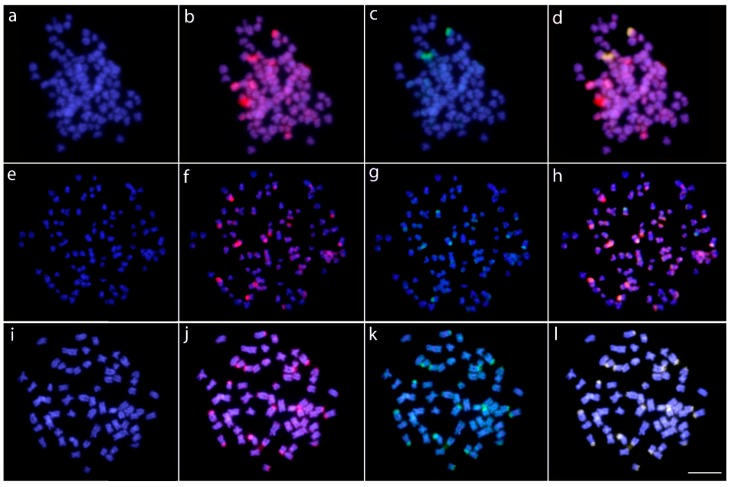
Metaphase chromosome spreads of *Kryptopterus geminus* (**a**–**d**), *Wallago attu* (**e**–**h**), and *Phalacronotus bleekeri* (**i**–**l**) after comparative genomic hybridization (CGH) procedures. Male-derived genomic probes from *K. geminus* and K. limpok were hybridized together against male chromosomes of *K. geminus* (**a**–**d**). Male-derived genomic probes from *W. attu* and *W. micropogon* were hybridized together against male chromosomes of *W. attu* (**e**–**h**). Male-derived genomic probes from *P. bleekeri* and *P. apogon* were hybridized together against male chromosomes of *P. bleekeri* (**i**–**l**). First column (**a**,**e**,**i**): DAPI images (blue). Second column (**b**,**f**,**j**): hybridization pattern using *K. geminus* (**b**), *W. attu* (**f**), and *P. bleekeri* (**j**) gDNA probes (red). Third column (**c**,**g**,**k**): hybridization pattern using *K. limpok* (**c**), *W. micropogon* (**g**), and *P. apogon* (**k**) gDNA probes (green). Fourth column (**d**,**h**,**l**): merged images of both genomic probes and DAPI staining. The shared genomic regions are depicted in yellow. Bar = 5 μm.

CGH experiments employing the gDNA of *Kryptopterus* (*K. geminus x K. limpok*) and *Wallago* (*W. attu x W. micropogon*) indicated a large genomic divergence between the congeneric species. Specifically, *K. geminus* and *W. attu* exhibited many hybridization sites in the centromeric and terminal chromosomal regions when their own gDNA probes were hybridized against their chromosomal background. However, the *K. limpok* and *W. micropogon* gDNA probes produced only some weak terminal signals when hybridized against the *K. geminus* and *W. attu* chromosomes, respectively. In contrast, the *Phalacronotus* species (*P. bleekeri* and *P. apogon*) showed a significant shared repetitive content (Figure 6).

## 3. Discussion

The comparison of cytogenetic data for silurid species uncovered a large genomic diversification. This has been highlighted by some published data (Table 1), as well as here by highly diversified 2*n*, karyotype structures, numbers, and positions of ribosomal genes, and likely also by genomic differentiation, as preliminarily demonstrated by CGH experiments. The review of available cytotaxonomic data indicated a remarkable karyotype diversity, where the 2*n* number ranges from 28 in *S. microdorsalis* to 92 in *Kryptopterus cryptopterus* and *K. geminus* (Table 1). Oliveira and Gosztonyi [68] suggested that 2*n* = 56 corresponds to the typical number of chromosomes for Siluriformes, as this same number is found in *Diplomystes*, a sister group of all extant Siluriformes [69], in addition to 2*n* = 54–58 being the most frequent pattern among siluriform catfishes. The extensive numerical variation of the chromosome number, both below and far above the supposedly basal 2*n*, as well as the rate of bi-armed chromosomes in the karyotypes, indicate that a diversified number of rearrangements including fissions, fusions, and inversions may have acted to give rise to karyotypic diversity noticed in this family.

What could have driven the extensive karyotype diversification among silurid species? 

It is widely known that karyotype diversification relates to speciation processes [70,71,72], sometimes with repetitive DNAs acting as primary driving forces (reviewed in the work of [73]). The mapping of repetitive sequences, especially ribosomal genes, has proven useful for estimating evolutionary karyotype changes [74]. Although rDNAs represent conservative elements of the eukaryotic genomes, recent studies have shown that the dynamism of the rDNA clusters is strongly related to significant intragenomic diversification [21,75,76,77,78,79,80,81]. Accordingly, rDNA elements showed remarkable differences among silurid species, especially regarding the high variability in the number and position of the 5S rDNA sites as compared with the more stable pattern of the 18S rDNA sites.

Extensive chromosomal variability of 5S rDNA loci also has been described for several other fish groups ([21,22,79,82]; for review, see the work of [74]). A question that arises is whether the dispersion of this rDNA class would be a byproduct of genomic/chromosomal changes. However, the absence of a direct correlation between higher 2*n* numbers and amplification and dispersion of the 5S rDNA clusters is an indication that this rDNA class was not the unique trigger for the chromosomal rearrangements occurring among the respective silurid species. In this sense, an alternative and attractive hypothesis refers to the action of transposable elements. Indeed, in several species, a significant fraction of the rDNA units is interrupted by transposable elements (TEs) highly specialized for insertions [82,83,84,85,86]. In some cases, TEs have been postulated to play a decisive role in spreading rDNA sequences over the genome [22,23,82]. Remarkably, structural changes in the location of rDNAs also could be linked with speciation events. In the sister salmonid species, *Coregonus albula* and *C. fontanae,* ecological speciation was directly associated with the spreading of rDNA sites, affecting recombination rates in both genomes [22]. It is known that multiple rDNA insertions in new genomic regions may create “hot spots” that promote chromosome rearrangements, representing a pathway for rapid genome reorganization during speciation (reviewed in the work of [84]). Nevertheless, up to now, we have no data concerning TEs among silurid species, which will be the goal of further investigations to assess this hypothesis. Besides, it is known that a variety of teleost lineages have undergone one or more rounds of independent whole-genome duplications (WGDs), which are among the most important evolutionary events occurring in fish species [18]. Although there is no direct indicative that silurids analyzed here have experienced WDG events, we cannot exclude the potential role of this process in the high genomic/chromosomal divergence observed.

The available data for silurids allow us to recognize three particular patterns in relation to the 2*n* numbers and karyotype structures that they present: (i) congeneric species that are highly divergent, as observed in *Kryptopterus* and *Wallago* species; (ii) congeneric species that share similar features, as represented by the two *Phalacronotus* species, *P. apogon*, and *P. bleekeri;* and (iii) particular species displaying a significantly lower chromosome number compared with the other species, as observed in *S. phaiosoma*. For the latter, although multiple chromosomal fusions would be expected to be related, no interstitial telomeric sequences (ITS) were observed (Figure S1). However, this does not definitely refute the possibility that fusion events occurred during karyotypic diversification, as losses of telomeric sequences can occur after such rearrangements, leading to gradual shortening of non-functional telomeric arrays [87,88,89].

**Figure 7:**
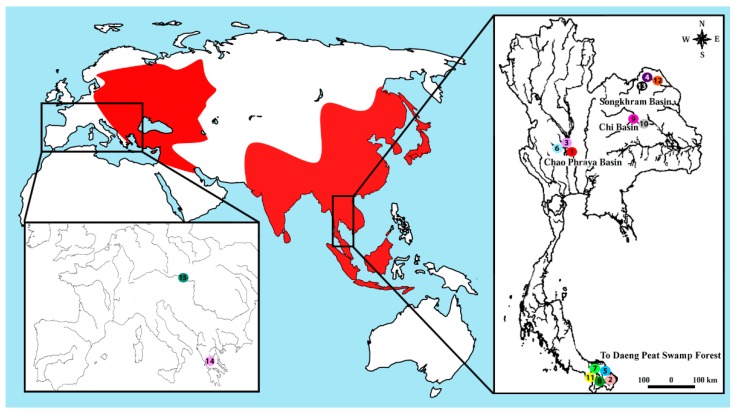
The current distribution of the fish family Siluridae (red color). Inset: Thailand map indicating the collection sites of the 13 species studied herein. **1**. *Belodontichthys truncates* (red circle); **2**. *Kryptopterus bicirrhis* (light pink circle); **3**. *Kryptopterus giminus* (violet circle); **4**. *Kryptopterus limpok* (purple circle); **5**. *Kryptopterus microcephalus* (blue circle); **6**. *Micronema cheveyi* (light blue circle); **7**. *Ompok fumidus* (light green circle); **8***. Ompok siluroides* (green circle); **9**. *Phalacronotus apogon* (pink circle); **10**. *Phalacronotus bleekeri* (grey circle); **11**. *Silurichthys phaiosoma* (yellow circle); **12**. *Wallago attu* (orange circle); **13**. *Wallago micropogon* (black circle); **14**. *Silurus aristotelis* (pink circle); and **15**. *Silurus glanis* (dark green circle). The maps were created using QGis 3.4.3, Inkscape 0.92 and Photoshop 7.0.

Regarding the first two above-mentioned scenarios, we performed CGH experiments in order to assess whether they are linked with the repetitive DNA content. The remarkable chromosomal dynamism in both *Kryptopterus* and *Wallago* species corresponds with an extensive variation of their repetitive DNA content, as demonstrated by a range of non-overlapping species-specific signals revealing an advanced stage of sequence divergence among their genomes (Figure 7). In fact, such repetitive DNA differentiations occurred concomitantly with 2*n* and structural changes in karyotypes. In contrast, no substantial variation of repetitive DNA content was found among the *Phalacronotus* species, where the hybridization of both gDNAs produced no species-specific signal amplifications (Figure 6). In these species, karyotypic changes were markedly reduced. As repetitive DNAs are highly abundant in eukaryotic genomes and display faster evolutionary rates [19,90,91], their role as the main factor in promoting karyotype rearrangements has been extensively investigated. Several reports have evidenced huge inter-population variations of this genomic fraction, promoting biodiversity and possibly linked with ongoing speciation and differentiation of sex-specific regions [24,92,93,94,95].

Other siluriform groups also experienced massive karyotype differentiation. *Clarias* species (Clariidae), for example, display a large range of 2*n* number, from 48 to 104 [79,96], in some cases also including polyploidization and interspecific hybridization events. In *C. batrachus* (2*n* = 104), a surprising spread of the 5S rDNA sequences over 27 chromosomal pairs occurs, directly linked with multiple centric fissions [79]. In addition, some other siluriform lineages experienced large karyotypic differentiation such as Callichthyidae, Loricariidae, and Trichomycteridae. In contrast, in families Amyblicipitidae, Ictaluridae, and Sisoridae, only few species possess a reduced 2*n* number (reviewed in the work of [97]). However, it is evident that siluriform catfishes have, in general, much higher karyotypic diversity than their sister lineage Characiformes. With caution, in view of the fact that only about 15% of the siluriform fishes have been cytogenetically examined to date, it is noteworthy that the largest chromosomal diversity was observed for Siluridae.

## 4. Materials and Methods

### 4.1. Individuals and Mitotic Chromosome Preparation

Fifteen silurid species were collected from distinct natural ecosystems of Thailand and Europe (Figure 7). The numbers and sexes of the individuals are presented in Table 2. The specimens were deposited in the fish collections of the Cytogenetic Laboratory, Department of Biology, Faculty of Science (Khon Kaen University) and National Museum of Natural History, Paris (MNHN 1997–0481, MNHN 1996–1382). Mitotic chromosomes were obtained by the protocol described in the work of [98]. All the experiments followed ethical protocols, and anesthesia was conducted with clove oil prior to the sacrifice of the animals. The process was approved by the Animal Ethics Committee of Khon Kaen University based on the Ethics of Animal Experimentation of the National Research Council of Thailand AEKKU23/2558. Samples of *S. glanis* and *S. aristotelis* were obtained under state fisheries permits and research was conducted with approval from the University of Thessaloniki Ethics Committee.

### 4.2. Fluorescence In Situ Hybridization (FISH)

FISH was done under high-stringency conditions on metaphase chromosome spreads [99], with specific probes for 5S and 18S rDNA and telomeric sequences. The 5S rDNA probe included the transcriptional segment of the 5S rRNA gene, with 120 base pairs (bp), and the 200-base pair non-transcribed spacer (NTS) [100]. The 18S rDNA probe corresponded to a 1400 base-pair segment of the 18S rDNA gene [101]. Both rDNA probes were directly labeled with the Nick-translation Labeling Kit (Jena Bioscience, Jena, Germany) by the fluorescent labels Atto488 (18S rDNA) and Atto550 (5S rDNA), according to the manufacturer’s manual. We applied both rDNA probes in all analyzed species, with the exception of S. aristotelis, where only 18S rDNA mapping was performed. 

In order to check the presence of ITS (interstitial telomeric sequences), telomeric (TTAGGG)*_n_* sequences were mapped in the species with the lowest 2*n* (*S. phaiosoma*) using the DAKO Telomere PNA FISH Kit/Cy3 (DAKO, Glostrup, Denmark).

### 4.3. Comparative Genome Hybridization (CGH)

Total genomic DNA (gDNAs) of the *K. geminus*, *K. limpok*, *P. apogon*, *P. bleekeri, W. attu*, and *W. micropogon* were extracted from liver tissue by the standard phenol-chloroform-isoamyl alcohol method [102]. As substantial variation in both 2*n* number and karyotype formula were observed among species of the genus *Kryptopterus* and *Wallago*, the gDNA of *K. geminus* was compared with that of *K. limpok* in metaphase chromosomes of *K. geminus.* Similarly, the gDNAs of *W. attu* and *W. micropogon* were hybridized in metaphase chromosomes of *W. attu.* For these purposes, gDNAs of *K. geminus* and *W. attu* were directly labeled with Atto550 using the Nick-translation Labeling Kit (Jena Bioscience, Jena, Germany), while the gDNAs of *K. limpok* and W. micropogon were labeled with Atto488. To block common genomic repetitive sequences, C0t-1 DNA (i.e., a fraction of genomic DNA enriched for highly and moderately repetitive sequences), prepared according to Zwick et al. [103], was used in all experiments. The final hybridization mixture for each experiment was composed of 500 ng labeled DNA of each compared species, plus 15 μg of male-derived C0t-1 DNA from the respective species and the hybridization buffer (50% formamide, 2× SSC, 10% SDSC 10% dextran sulfate and Denhardt’s solution, pH 7.0). The gDNA of *Phalacronotus apogon* (Atto488) was also compared with that of *P. bleekeri* (Atto550) against metaphase chromosomes of *P. apogon*. The CGH experiments were performed according to Symonová et al. [22].

### 4.4. Cytogenetic Analyses

At least 30 metaphase spreads per individual were analyzed to confirm the 2*n*, karyotype structure, and FISH results. Images were captured using an Axioplan II microscope (Carl Zeiss Jena GmbH, Germany) with CoolSNAP and the images were processed using Image Pro Plus 4.1 software (Media Cybernetics, Silver Spring, MD, USA). Chromosomes were classified as metacentric (m), submetacentric (sm), subtelocentric (st), or acrocentric (a), according to the arm length ratios [104].

## 5. Conclusions

Chromosomal characteristics, including the mapping of repetitive DNA sequences and CGH procedures, clarified the evolutionary dynamism among silurid species. In this sense, the known extensive diversification of their karyotypic macrostructure could be better characterized. Our data provide evidence for a direct correlation between the genomic repetitive content and the notable karyotypic divergence in silurids. Thus, it is likely that repetitive DNAs played a direct role in promoting the chromosomal differentiation and biodiversity within this fish family.

## Figures and Tables

**Table 1:** Review of cytogenetic data in the family Siluridae. The species now analyzed are highlighted.

Species	Locality	2*n*	NF	Karyotype	NORs/18S rDNA	Reference
*Belodontichthys truncatus*	Thailand	62	100	20m+10sm+8st+24a	-	[29]
*B. truncatus*	Thailand	62	112	14m+30sm+6a+12t	2	[30]
*B. truncatus*	Thailand	62	106	14m+30sm+18st/a	2	Present work
*Kryptopterus bicirrhis*	SE Asia	60	-	-	-	[31]
*K. bicirrhis*	Thailand	64	98	20m+10sm+4st+30a	-	[29]
*K. bicirrhis*	Thailand	64	96	14m+18sm+32st/a	2	Present work
*K. cryptopterus*	Thailand	92	110	8m+10st+74a	-	[28]
*K. geminus*	Thailand	92	102	6m+4sm+82st/a	4	Present work
*K. limpok*	Thailand	60	86	12m+14sm+34st/a	2	Present work
*K. macrocephalus*	Thailand	62	98	24m+12sm+26a	-	[32]
*K. macrocephalus*	Thailand	62	72	4m+6sm+52st/a	2	Present work
*Micronema cheveyi*	Thailand	78	96	4m+6sm+10a+58t	2	[30]
*M. cheveyi*	Thailand	78	96	4m+6sm+10a+58t	2	[33]
*M. cheveyi*	Thailand	78	88	4m+6sm+68st/a	2	Present work
*Ompok bimaculatus*	India	42	72	18m+12sm+12a (F) XX	-	[34]
*O. bimaculatus*	India	41	70	17m+12sm+12a (M) XY	-	[34]
*O. bimaculatus*	India	42	72	6m+24sm+12a (F)	2	[35]
*O. bimaculatus*	India	41	70	5m+24sm+12a (M)	2	[35]
*O. bimaculatus*	India	42	68	12m+14sm+16a/t	-	[36]
*O. bimaculatus*	India	54	102	16m+26sm+6a+6t	2	[26]
*O. bimaculatus*	Thailand	50	90	14m+20sm+6a+10t	2	[37]
*O. fumidus*	Thailand	60	82	20m+2sm+2st+36a	-	[32]
*O. fumidus*	Thailand	64	88	10m+10sm+44st/a	2	Present work
*O. pabda*	India	54	100	28m+10sm+8st+8a	-	[38]
*O. pabda*	India	42	-	-	-	[39]
*O. pabda*	India	42	68	12m+14sm+16a/t	-	[36]
*O. pabda*	India	42	84	26m+10sm+6a	2	[26]
*O. pabo*	India	54	102	36m+12sm+6a	-	[40]
*O. siluriodes*	Thailand	50	88	34m+2sm+2st+12a	-	[28]
*O. siluriodes*	Thailand	50	96	20m+22sm+4a+4t	2	[30]
*O. siluriodes*	Thailand	50	92	20m+22sm+8st/a	4	Present work
*Phalacronotus apogon*	Thailand	64	108	18m+20sm+6a+20t	2	[41]
*P. apogon*	Thailand	64	102	18m+20sm+26st/a	2	Present work
*P. bleekeri*	Thailand	64	92	20m+6sm+2st+36a	-	[28]
*P. bleekeri*	Thailand	64	106	14m+22sm+6a+22t	2	[33]
*P. bleekeri*	Thailand	64	102	18m+20sm+26st/a	2	Present work
*Silurichthys phaiosoma*	Thailand	40	46	2m+4sm+8st+26a	-	[32]
*S. phaiosoma*	Thailand	40	42	2m+38st/a	10	Present work
*S. schneideri*	Thailand	40	50	6m+4sm+4st+26a	-	[32]
*Silurus aristotelis*	Greece	58	116	30m+20sm+8st	-	[42]
*Si. aristotelis*	Greece	58	102	20m+24sm+14st/a	2	[43]
*Si. aristotelis*	Czech	58	104	22m+24sm+12st/a	2	Present work
*Si. asotus*	-	58	-	58t	-	[44]
*Si. asotus*	Japan	58	104	38m/sm+8st+12a	-	[45]
*Si. asotus*	Japan	58	104	46m/sm+12st/a	2	[46]
*Si. asotus*	Japan	58	102	44m/sm+14st/a	-	[47,48]
*Si. asotus*	Korea	58	106	24m+24sm+10st/a	-	[49]
*Si. asotus*	China	58	102	20m+24sm+10st+4a	-	[50,51,52]
*Si. asotus*	China	58	98	20m+14sm+6st+18a	2	[53,54]
*Si. asotus*	Mongolia	58	-	42m/sm+16st/a	-	[27]
*Si. asotus*	Korea	58	106	-	-	[55]
*Si. asotus*	China	58	112	20m+24sm+10st+4a	-	[56]
*Si. biwaensis*	Japan	58	102	44m/sm+14st/a	-	[47]
*Si. glanis*	-	60	100	40m/sm/st+20a	-	[57]
*Si. glanis*	-	60	100	40m/sm+20a	-	[58]
*Si. glanis*	Czech	60	120	28m+26sm+6st	-	[59]
*Si. glanis*	-	60	98	38m/sm+22a	-	[60]
*Si. glanis*	Serbia	60	94	16m+18sm+14st+12a	-	[61]
*Si. glanis*	Russia	60	110	18m+32sm/st+10a	-	[62]
*Si. glanis*	Serbia	48	78	30m/sm+18st/a	-	[63]
*Si. glanis*	Czech	60	120	22m+38sm/st	2	[64]
*Si. glanis*	Czech	60	106	22m+24sm+14st/a	2	Present work
*Si. lithophilus*	Japan	58	102	44m/sm+14st/a	-	[47]
*Si. lithophilus*	China	58	98	20m+20sm+18st/a	-	[50]
*Si. meridionalis*	China	58	112	20m+20sm+14st+4a	-	[50,51,52]
*Si. meridionalis*	Korea	60	106	22m+24sm+12st/a+2 microchromosomes	-	[49]
*Si. microdorsalis*	Korea	28	56	12m+14sm+2st	-	[27]
*Si. soldatovi*	China	58	112	24m+16sm+14st+4a	-	[65]
*Wallago attu*	India	86	106	12m+6sm+2st+66a	-	[66]
*W. attu*	India	86	116	10m+12sm+8st+56a	-	[67]
*W. attu*	Thailand	88	110	16m+2sm+4st+66a	-	[29]
*W. attu*	Thailand	88	108	6m+6sm+8a+68t	2	[30]
*W. attu*	Thailand	88	100	6m+6sm+76st/a	2	Present work
*W. micropogon*	Thailand	56	86	26m+4sm+26a	-	[29]
*W. micropogon*	Thailand	56	100	18m+20sm+6a+12t	2	[30]
*W* *. micropogon*	Thailand	56	94	18m+20sm+18st/a	4	Present work

* NF = fundamental number; 2*n* = diploid number; M = male; F = female; and NOR = nucleolar organizer region.

**Table 2:** Collection sites for the analyzed species with the respective sample sizes.

Species	Locality	**No. of Individuals**
*Belodontichthys truncatus*	Chao Phraya Basin (Thailand) (site 1)	(04 ♀; 04 ♂)
*Kryptopterus bicirrhis*	To Daeng peat swamp forest (Thailand) (site 2)	(07 ♀; 08 ♂)
*Kryptopterus geminus*	Chao Phraya Basin (Thailand) (site 3)	(08 ♀; 11 ♂)
*Kryptopterus limpok*	Songkhram Basin (Thailand) (site 4)	(07 ♀; 10 ♂)
*Kryptopterus macrocephalus*	To Daeng peat swamp forest (Thailand) (site 5)	(06 ♀; 06 ♂)
*Micronema cheveyi*	Chao Phraya Basin (Thailand) (site 6)	(09 ♀; 10 ♂)
*Ompok fumidus*	To Daeng peat swamp forest (Thailand) (site 7)	(05 ♀; 07 ♂)
*Ompok siluroides*	To Daeng peat swamp forest (Thailand) (site 8)	(04 ♀; 05 ♂)
*Phalacronotus apogon*	Chi Basin (Thailand) (site 9)	(06 ♀; 05 ♂)
*Phalacronotus bleekeri*	Chi Basin (Thailand) (site 10)	(07 ♀; 04 ♂)
*Silurichthys phaiosoma*	To Daeng peat swamp forest (Thailand) (site 11)	(04 ♀; 06 ♂)
*Wallago attu*	Songkhram Basin (Thailand) (site 12)	(03 ♀; 04 ♂)
*Wallago micropogon*	Songkhram Basin (Thailand) (site 13)	(04 ♀; 04 ♂)
*Silurus aristotelis*	Trichonida Lake (Greece) (site 14)	(03 ♀; 05 ♂)
*Silurus glanis*	Dyje River, Danube basin (Czech republic) (site 15)	(08 ♀; 06 ♂)

Sites 1 to 15 correspond to the localization of each collection region shown in Figure 7.

## Supplementary Materials

The following are available online at https://www.mdpi.com/1422-0067/20/14/3545/s1, Figure S1: Metaphase plates of *Silurichthys phaiosoma* showing the location of telomeric (TTAGGG)*_n_* repeats. Bar = 5 μm.
