# Supplementary material for: Genomic Organization of Repetitive DNA Elements and Extensive Karyotype Diversity of Silurid Catfishes (Teleostei: Siluriformes): A Comparative Cytogenetic Approach
Source: Int J Mol Sci. 2019 Jul 19;20(14):3545. doi: 10.3390/ijms20143545 (PMC6678683; doi:10.3390/ijms20143545)
Supplement: Supplementary file 1 [file ijms-20-03545-s001.pdf]

Supplementary

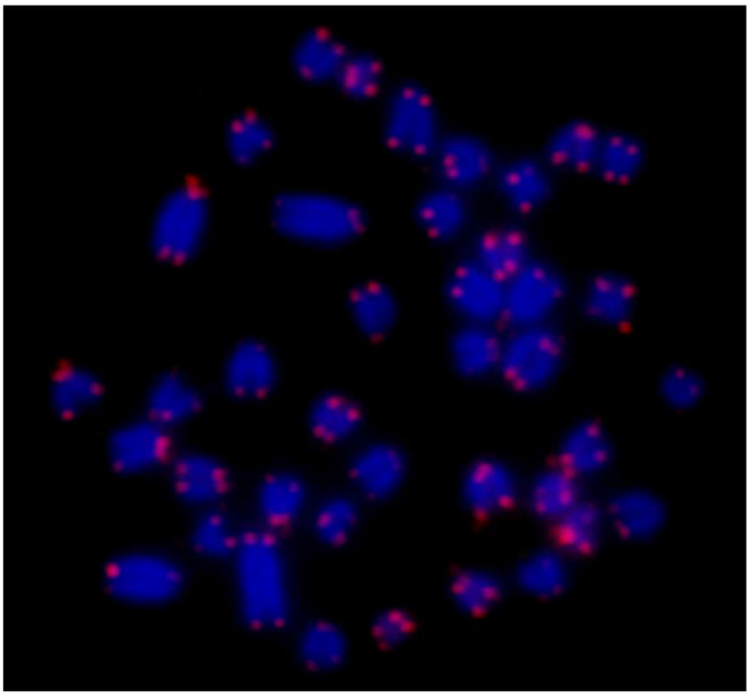

Figure S1. Metaphase plates of *Silurichthys phaiosoma* showing the location of telomeric (TTAGGG)*n* repeats. Bar = 5 μm.
